# sCD28, sCD80, sCTLA-4, and sBTLA Are Promising Markers in Diagnostic and Therapeutic Approaches for Aseptic Loosening and Periprosthetic Joint Infection

**DOI:** 10.3389/fimmu.2021.687065

**Published:** 2021-08-06

**Authors:** Jil M. Jubel, Thomas M. Randau, Janine Becker-Gotot, Sebastian Scheidt, Matthias D. Wimmer, Hendrik Kohlhof, Christof Burger, Dieter C. Wirtz, Frank A. Schildberg

**Affiliations:** ^1^Clinic for Orthopedics and Trauma Surgery, University Hospital Bonn, Bonn, Germany; ^2^Institute of Experimental Immunology, University Hospital Bonn, Bonn, Germany

**Keywords:** aseptic loosening, periprosthetic joint infection, immunoregulatory markers, sCD28, sCD80, sCTLA-4, sBTLA, osteoimmunology

## Abstract

Aseptic prosthetic loosening and periprosthetic joint infections (PJI) are among the most frequent complications after total knee/hip joint arthroplasty (TJA). Current research efforts focus on understanding the involvement of the immune system in these frequent complications. Different immune cell types have already been implicated in aseptic prosthetic loosening and PJI. The aim of this study was to systematically analyze aspirates from knee and hip joints, evaluating the qualitative and quantitative composition of soluble immunoregulatory markers, with a focus on co-inhibitory and co-stimulatory markers. It has been shown that these molecules play important roles in immune regulation in cancer and chronic infectious diseases, but they have not been investigated in the context of joint replacement. For this purpose, aspirates from control joints (i.e., native joints without implanted prostheses), joints with TJA (no signs of infection or aseptic loosening), joints with aseptic implant failure (AIF; i.e., aseptic loosening), and joints with PJI were collected. Fourteen soluble immunoregulatory markers were assessed using bead-based multiplex assays. In this study, it could be shown that the concentrations of the analyzed immunoregulatory molecules vary between control, TJA, AIF, and PJI joints. Comparing TJA patients to CO patients, sCD80 was significantly elevated. The marker sBTLA was significantly elevated in AIF joints compared to TJA joints. In addition, a significant difference for eight markers could be shown when comparing the AIF and CO groups (sCD27, sCTLA-4, sCD137, sCD80, sCD28, sTIM-3, sPD-1, sBTLA). A significant difference was also reached for nine soluble markers when the PJI and CO groups were compared (sLAG-3, sCTLA-4, sCD27, sCD80, sCD28, sTIM-3, sPD-1, IDO, sBTLA). In summary, the analyzed immunoregulatory markers could be useful for diagnostic purposes as well as to develop new therapeutic approaches for AIF and PJI.

## Introduction

In recent years, the continuously aging population and associated age-related morbidity have led to a marked increase in the number of implanted joint endoprostheses (1–3). As a result, an increase in consequent complications has been observed (2). These primarily include aseptic prosthetic loosening (referred to in this study as aseptic implant failure (AIF)) and peri-implant fractures, and also prosthetic joint infections (PJI), which can lead to septic prosthetic loosening (4–6). These typical complications can lead to significant limitations in daily activities due to pain, immobility, and chronic infections (7).

Current research efforts focus on understanding the immune system involvement, both qualitatively and quantitatively, in these frequent complications. Specific cell types of the immune system have been implicated in AIF (aseptic loosening) and PJI. As important mediators of osteolysis, macrophages play a significant role in the former complication (5, 8).

A new field of intensive research is the regulation of the immune system by immunoregulatory molecules, with a particular focus on so-called checkpoint molecules. These checkpoint molecules play an important role in immune regulation in cancer and chronic infectious diseases (9–12). At first, it was assumed that the main cells that are influenced by these molecules are T cells (10, 13–15). It is now known that these immunoregulatory markers also regulate other immune cells, such as macrophages, monocytes, and B cells (16, 17). Checkpoint molecules can be classified into co-stimulatory and co-inhibitory molecules. Co-stimulatory molecules, such as cluster of differentiation 27 (CD27), CD28, and glucocorticoid-induced TNFR-related protein (GITR), enhance the T cell response, while co-inhibitory molecules, such as programmed cell death protein 1 (PD-1) or cytotoxic T-lymphocyte-associated protein 4 (CLTA-4), reduce it (10, 18–20). Recently, soluble forms of checkpoint molecules, such as sPD-1 (soluble PD-1), sPD-L2 (soluble PD-L2), and sCTLA-4 (soluble CTLA-4) were found (21). Their role is not yet understood but first studies have shown that these soluble forms of checkpoint molecules can be involved in positive or negative immune regulation. Furthermore, the development, prognosis, and treatment of cancer (lung, gastric or renal cell cancer) and infectious diseases (hepatitis B) may be affected by changes in the plasma levels of soluble immune markers (22–25).

A recent PubMed query showed that there has not yet been a systematic evaluation of the associations between the concentrations of soluble immunoregulatory molecules and joint implant-associated complications.

Differentiation between aseptic and septic joint inflammation is difficult. Neither clinical nor biochemical markers can distinguish abacterial from bacterial joint inflammation, despite assessment of markers obtained from clinical examinations, blood and joint aspirate analyses, and microbiological and histological tissue analyses (26). For example, a negative bacterial result in a joint aspirate does not reliably exclude a bacterial infection (27, 28). Currently, different scoring systems are used to diagnose a PJI. MSIS criteria, for example, consist of several major and minor parameters. Based on this scoring system, a PJI is diagnosed when one out of two major criteria (two microorganism-positive cultures indicating the same pathogen; sinus tract communicating with the prosthesis) or three out of five minor criteria are fulfilled (CRP >10 mg/L; joint aspirate: leukocytes >3000 cells/μL, neutrophils >85%; single microorganism-positive tissue/aspirate sample; positive histology).

It is crucial to diagnose PJI early, differentiating septic from aseptic implant loosening so that specific therapy can be initiated at an early stage (29). If inflammation is treated inadequately, irreversible joint damage can occur, such as cartilage destruction with subsequent arthrosis and ankylosis (30). These changes can lead to functional loss of the affected joint and thus to permanent disabilities that affect everyday life. If the PJI progresses, the “ultima ratio” is amputation of the affected limb to save the patient’s life (31, 32).

The aim of this study was to systematically analyze aspirates from knee and hip joints, evaluating the qualitative and quantitative composition of soluble immunoregulatory markers for evaluating their potential as disease markers. For this purpose, aspirates from control (CO) joints (i.e., native joints without implanted prostheses), joints with total joint arthroplasty (TJA, i.e., fixed prostheses), joints with aseptic implant failure (AIF; i.e., aseptic loosening), and joints with periprosthetic joint infection (PJI) were evaluated and compared. The working hypothesis was that the qualitative and quantitative composition of soluble immunoregulatory molecules exhibits specific variations in aspirates from control, TJA, AIF, and PJI joints. Furthermore, one or more biomarkers may be specific for AIF or PJI. The identification of such biomarkers could lead to a better understanding of the pathomechanisms and new diagnostic and therapeutic approaches.

## Material and Methods

### Study Population

Consecutive patients (n = 99) treated between 2016 and 2019 at the Clinic for Orthopedics and Trauma Surgery of the University Hospital Bonn, Germany, were recruited. The patients were aged 18–100 years and had undergone synovial fluid aspiration for diagnostic or therapeutic purposes. Patients with sepsis or extra-articular infection were excluded. The included patients were divided into four groups: control (CO) patients with native joints (no prosthesis and no signs of infection); patients with fixed TJA (no signs of infection or aseptic loosening); patients with AIF (i.e., aseptic loosening); and patients with PJI. The ethics committee of the University of Bonn, Germany, approved the study, which was conducted according to the approved guidelines and the Helsinki Declaration.

### Classification

The classification developed by the Musculoskeletal Infection Society (MSIS) was used to identify patients with PJI. PJI was diagnosed when one major criterion (out of two major criteria) or three minor criteria (out of five minor criteria) were fulfilled. The major criteria are two microorganism-positive cultures (based on aspirate/tissue samples), indicating the same pathogen and sinus tract communicating with the prosthesis. The minor criteria are CRP >10 mg/L, leukocytes >3000 cells/μL of joint aspirate, neutrophils >85% in joint aspirate, single microorganism-positive tissue/aspirate sample, and positive histology. The diagnosis of AIF (aseptic loosening) was determined based on the MSIS criteria, clinical examination, and radiological signs.

### Data Collection

Data were collected on patient gender, age, BMI, and comorbidities (**Table 1**). In addition, laboratory results such as serum C-reactive protein (CRP), preoperative blood leukocyte counts, joint aspirate cell counts, intraoperative findings, sonication microbiology, and histopathology results were obtained from the medical records. All data were recorded in Microsoft Excel (Microsoft Corporation, Redmond, WA, USA).

**Table 1:** Patient characteristics (n = 99).

Variable	Overall (n = 99)	CO (n = 13)	TJA (n = 23)	AIF (n = 24)	PJI (n = 39)
**Age (year)**	67 ± 13	48 ± 13	65 ± 10	72 ± 9	71 ± 12
**Gender (m:f)**	39:60	7:6	10:13	7:17	15:24
**Hip**	35	2	6	11	16
**Knee**	64	11	17	13	23
**BMI (kg/m^2^)**	32 ± 8	26 ± 4	33 ± 6	32 ± 6	33 ± 11
**Diabetes mellitus**	23	1	3	2	17
**Rheumatoid arthritis**	10	1	3	2	4
**CRP (mg/dl)**	46 ± 106	2.4 ± 0.7	14 ± 20	8.9 ± 8.9	92 ± 24
**Leukocytes (10^9^/l)**	8.6 ± 3	8.5 ± 0.8	7.6 ± 1.6	8.2 ± 2.9	9 ± 0.6

Data are presented as mean ± standard deviation or as frequency. Patients with a prothesis were in general older than control patients without one. PJI patients had the highest concentration of CRP and leukocytes. CO, control group; TJA, total joint arthroplasty; AIF, aseptic implant failure; PJI, periprosthetic joint infection; BMI, body mass index; CRP, C-reactive protein.

### Aspirate Sample Collection

Preoperative or intraoperative hip or knee joint aspirates had previously been obtained during diagnostic or therapeutic procedures, and the material not used for clinical diagnostics was utilized in this study. Each synovial fluid sample was centrifuged at 1200 rpm for 10 min (Centrifuge 5810 R; Eppendorf AG, Hamburg, Germany) to remove the cellular components. The resulting supernatant was transferred in 0.5-mL aliquots and stored at -80°C.

### Bead-Based Multiplex Assays

Various soluble cytokines were measured using Immuno-Oncology Checkpoint 14-plex ProcartaPlex™ bead-based assays (ThermoFisher, Waltham, MA, USA) according to the manufacturer’s instructions. The detected soluble targets were: B- and T-lymphocyte attenuator (BTLA), glucocorticoid-induced TNFR-related protein (GITR), herpesvirus entry mediator (HVEM), indolamin-2,3-dioxygenase (IDO), lymphocyte-activation gene 3 (LAG-3), programmed cell death protein 1 (PD-1), programmed cell death 1 ligand 1 (PD-L1), programmed cell death 1 ligand 2 (PD-L2), T-cell immunoglobulin and mucin-domain containing-3 (TIM-3), cluster of differentiation 28 (CD28), CD80, CD137, CD27, and cytotoxic T-lymphocyte-associated protein 4 (CTLA-4). All samples were immediately thawed before conducting the assay. The wells were prewetted with 10 μL reading buffer and the antibody-labelled magnetic beads were vortexed for 30 s. Next, 12.5 μL of the beads was added to each well. After washing the wells, 12.5 μL of samples (or standards provided with the assay kit) was added to the beads in the wells and incubated in the dark for 120 min at room temperature, with shaking. The beads were then washed twice. Next, 6.25 μL detection antibody mixture was added to each well and incubated in the dark for another 30 min, with shaking. After washing the beads, 12.5 μL streptavidin-phycoerythrin (PE) was added to the wells and the beads were again incubated in the dark for 30 min, with shaking. Following another washing step, the beads were resuspended in 50 μL reading buffer for 5 min, with shaking. Finally, data were acquired using a Flexmap 3D^®^ system (Luminex Corporation, Austin, TX, USA). The raw data were transferred to a Microsoft Excel (Microsoft Corporation, Redmond, WA, USA) table for further analysis.

### Statistical Analysis

SPSS version 27 (IBM Corp., Armonk, NY, USA) was used for statistical analysis. The Kolmogorov–Smirnova test was performed to assess normality. The Kruskal-Wallis test was used to evaluate the statistical significance of the differences among the four groups. The Dunn’s Test was performed as post-hoc-test. The level of significance was set at p < 0.05 (* < 0.05, ** < 0.01, *** < 0.001). Descriptive statistics were calculated using GraphPad Prism 9 (GraphPad Software, La Jolla, CA, USA). All results are presented using boxplots showing the median and interquartile range (IQR).

## Results

The qualitative and quantitative composition of soluble immunoregulatory markers, focusing on co-inhibitory and co-stimulatory markers, was evaluated. Aspirates from control joints, joints with fixed TJA (no signs of infection or aseptic loosening), joints with AIF (aseptic loosening), and joints with PJI were compared.

Patient information is presented in **Table 1**. The overall male-to-female ratio was 2:3. The control patients were younger (mean age 48 years) than the patients with TJA (mean age 65 years) and the patients with periprosthetic complications (AIF: mean age 72 years, PJI: mean age 71 years). Furthermore, the control patients had a lower BMI (26 kg/m^2^) than the patients in the other three cohorts (TJA: 33 kg/m^2^, AIF: 32 kg/m^2^, PJI: 33 kg/m^2^). Preoperative routine blood analysis showed an increased level of CRP for PJI patients (92 mg/dl), and 44% of the PJI patients had diabetes mellitus.

**Table 2** shows the mean concentrations of the 14 soluble immunoregulatory markers from hip and knee aspirates (based on bead-based multiplex assays) in each of the four groups. Overall, the control patients tended to have the lowest mean concentrations of immunoregulatory markers. TJA patients tended to have higher mean concentrations than control patients, while AIF patients tended to have higher concentrations than TJA patients. For many markers, the highest mean concentration was found in PJI patients, though sBTLA, sCD80, and sCD27 were higher in AIF patients than PJI patients (**Table 2**).

**Table 2:** Mean concentrations according to bead-based multiplex assays.

Marker	CO (pg/ml)	TJA (pg/ml)	AIF (pg/ml)	PJI (pg/ml)
**sCTLA-4**	*59.33 ± 16.92*	222.52 ± 73.29	236.29 ± 33.73	**450.03 ± 58.53**
**sPD-1**	*32.77 ± 15.32*	120.61 ± 35.10	171.75 ± 28.99	**253.74 ± 59.44**
**sPD-L1**	*15.31 ± 6.60*	40.83 ± 19.62	92.38 ± 21.37	**289.92 ± 116.94**
**sPD-L2**	9113.77 ± 2417. 93	*8808.78 ± 1953.44*	15516.71 ± 1977.11	**15966.54 ± 1948.67**
**sTIM-3**	*6534.31 ± 753.27*	8435.04 ± 931.20	10649.71 ± 743.64	**11032.33 ± 805.93**
**sLAG-3**	*168.15 ± 67.58*	208.61 ± 49.94	276.38 ± 46.48	**319.69 ± 38.40**
**sBTLA**	*594.92 ± 199.10*	2220.57 ± 1125.36	**4053.50 ± 818.41**	3716.62 ± 674.90
**sHVEM**	*13.31 ± 13.31*	140.00 ± 453.79	541.00 ± 2064.83	**896.97 ± 437.64**
**IDO**	*38.46 ± 16.09*	171.04 ± 83.37	675.17 ± 291.29	**1892.77 ± 519.09**
**sCD28**	*200.58 ± 167.92*	1956.35 ± 764.85	3300.63 ± 663.01	**4547.46 ± 717.21**
**sCD80**	*238.23 ± 66.18*	1661.00 ± 450.41	**1911.00 ± 195.57**	1671.92 ± 184.79
**sCD27**	*5610.23 ± 2444.59*	10570.39 ± 2774.63	**34988.13 ± 8975.34**	32088.36 ± 5436.80
**sGITR**	59.08 ± 24.95	*55.61 ± 21.63*	70.79 ± 15.44	**175.97 ± 46.54**
**sCD137**	*1832.83 ± 606.11*	11892.73 ± 3469.32	10649.00 ± 3051.80	**14389.70 ± 8378.15**

Concentrations (pg/ml) of 14 immunoregulatory markers in hip and knee joint aspirates in each of the four groups are expressed as mean ± standard deviation. The highest values in each row are indicated in bold and the lowest values in italic. CO, control group; TJA, total joint arthroplasty; AIF, aseptic implant failure; PJI, periprosthetic joint infection; s, soluble; CTLA-4, cytotoxic T-lymphocyte-associated protein 4; PD-1, programmed cell death protein 1; PD-L1, programmed cell death 1 ligand 1; PD-L2, programmed cell death 1 ligand 2; TIM-3, T-cell immunoglobulin and mucin-domain containing-3; LAG-3, lymphocyte-activation gene 3; BTLA, B- and T-lymphocyte attenuator; HVEM, herpesvirus entry mediator; IDO, indolamin-2,3-dioxygenase; GITR, glucocorticoid-induced TNFR-related protein; CD, cluster of differentiation.

**Figure 1:**
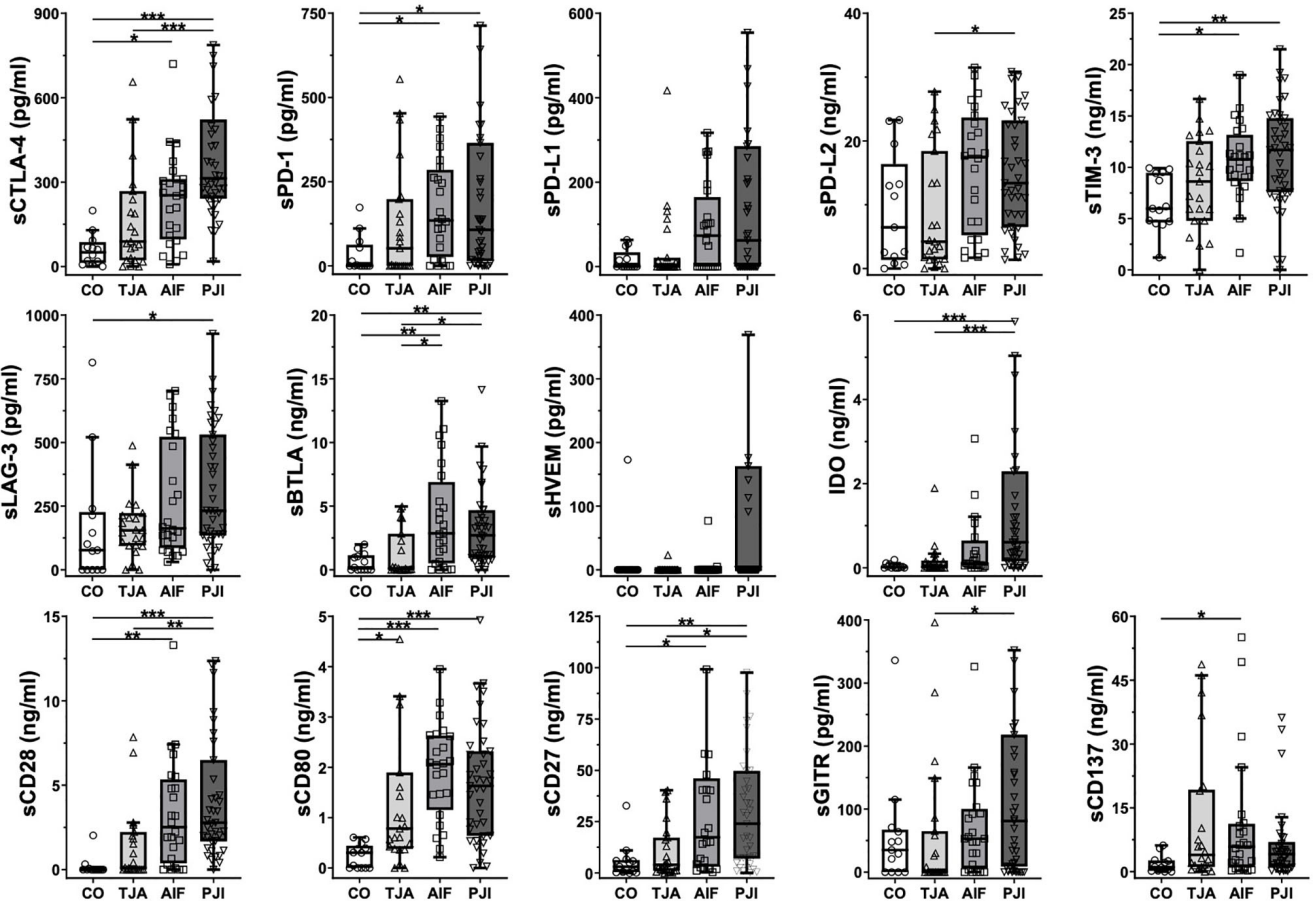
Boxplots of the analyzed immunoregulatory markers. Boxplots show the median and interquartile range for each marker. Concentrations are given in pg/ml or ng/ml. In general, the control (CO) patients tended to have the lowest concentrations, whereas the highest concentrations were measured in AIF (sPD-1, sPD-L1, sPD-L2, sBTLA, sCD80, and sCD137) or PJI (sCTLA-4, sTIM-3, sLAG-3, sHVEM, IDO, sCD28, sCD27, and sGITR) patients. CO, control group; TJA, total joint arthroplasty; AIF, aseptic implant failure; PJI, periprosthetic joint infection; s, soluble; CTLA-4, cytotoxic T-lymphocyte-associated protein 4; PD-1, programmed cell death protein 1; PD-L1, programmed cell death 1 ligand 1; PD-L2, programmed cell death 1 ligand 2; TIM-3, T-cell immunoglobulin and mucin-domain containing-3; LAG-3, lymphocyte-activation gene 3; BTLA, B- and T-lymphocyte attenuator; HVEM, herpesvirus entry mediator; IDO, indolamin-2,3-dioxygenase; GITR, glucocorticoid-induced TNFR-related protein; CD, cluster of differentiation. *p < 0.05, **p < 0.01, ***p < 0.001.

**Figure 1** shows boxplots of the data regarding all 14 immunoregulatory markers, expressed as the median and interquartile range (IQR). In general, the lowest median concentrations of the markers were found in the control patients, whereas the highest were found in either AIF patients (sPD-1, sPD-L1, sPD-L2, sBTLA, sCD80, and sCD137) or PJI patients (sCTLA-4, sTIM-3, sLAG-3, sHVEM, IDO, sCD28, sCD27, and sGITR).

Regarding the results of the Kruskal-Wallis test, TJA patients tended to have higher concentrations than control patients, with significant levels being reached for the co-stimulatory marker sCD80 (p = 0.015) (**Figure 1**). In addition, the majority of the concentrations were also significantly higher in AIF patients than in control patients, with sCD28, sCD80, and sBTLA reaching a significance of p < 0.01 (**Figure 1**). Moreover, differences in concentrations between the PJI and control groups were significant, with sCTLA-4, IDO, sCD28, and sCD80 reaching a significance of p < 0.001 (**Figure 1**).

As shown in **Table 2**, the mean concentrations of all immunoregulatory markers were higher in AIF patients than in TJA patients, except for sCD137 (TJA: 11892.73 pg/ml, AIF: 10649.00 pg/ml). For example, the mean concentration of the co-inhibitory markers PD-1 was 171.75 pg/ml in AIF patients and only 120.61 pg/ml in TJA patients. Regarding the results of the Kruskal-Wallis test concerning the median concentrations, significantly higher levels were found in AIF patients compared to TJA patients for sBTLA (p = 0.036) (**Figure 1**).

Additionally, the mean concentrations of all 14 immunoregulatory markers (sCTLA-4, sPD-1, sPD-L1, sPD-L2, sTIM-3, sLAG-3, sBTLA, sHVEM, IDO, sCD28, sCD80, sCD27, sGITR, and sCD137) were higher in PJI patients compared to TJA patients. Kruskal-Wallis tests analyzing the median concentrations showed that sCTLA-4 and IDO reached a significance of p < 0.001 (**Figure 1**).

The mean concentrations in PJI patients were generally higher than in AIF patients (**Table 2**). However, AIF patients had higher levels of sBTLA, sCD80, and sCD27 compared to PJI patients. Investigating the median concentrations, no significant differences could be detected (**Figure 1**).

Additionally, we performed supplemental analyses to compare the marker concentrations of hip aspirates in comparison to knee aspirates. No significant trends could be seen, except for sLAG-3 in the PJI group (**Supplementary Table 1**). We also investigated whether diabetes mellitus (DM) had an influence on the investigated markers. In this study, only patients with a DM type II were found. When comparing DM patients with non-DM patients, we could not detect any significant difference (**Supplementary Table 2**). Interesting was the fact that the majority of patients with DM were in the PJI group. Similarly, also no significant difference could be found when comparing patients with and without rheumatoid arthritis (RA) (**Supplementary Table 3**).

In summary, the concentrations of the measured immunoregulatory markers differed between control (native), TJA, AIF, and PJI joints. The lowest levels were generally found in control joints, followed by TJA joints. Higher concentrations were generally found in AIF joints and the highest concentrations were generally found in PJI joints.

## Discussion

According to Wengler et al., the number of primary hip arthroplasties in Germany increased by 10.9% to 155,300 per year between 2005 and 2011 (33). During the same period, primary knee arthroplasty procedures increased by 21.6% to 152,500 per year (33).

One complication of endoprosthesis is aseptic joint inflammation, which can lead to AIF (aseptic implant failure). Another complication is septic joint inflammation caused by a bacterial infection (PJI), which can lead to septic prosthetic loosening. Wooley et al. reported that aseptic prosthetic loosening occurs in 20–25% of endoprosthetic implants (34), and approximately 1–2% of primary implants become infected, according to Trampuz et al. (2). These complications play significant roles in routine clinical practice, as patients experience severe reductions in quality of life, and the costs of treatment burden the healthcare system (2, 35, 36).

The variations in human immune responses related to joint endoprostheses, AIF, and PJI are not yet fully understood. This study presents an analysis of various soluble immunoregulatory markers (focusing on checkpoint molecules) in joint aspirates obtained during diagnostic or therapeutic procedures.

Checkpoint molecules play an essential role in modulating immune cells (11, 18). They are best known for regulating T cells, though it is now recognized that other immune cells, such as macrophages and monocytes, are also controlled by these molecules (16, 17). T cells are part of the adaptive immune system, i.e., the acquired immune response that acts against specific pathogens. Antigen-presenting cells present previously phagocytosed antigens *via* the major histocompatibility complex (MHC) (37, 38). The MHC binds to the T cell receptor on T cells, which activates them (38–40). Parry et al. found that MHC-dependent antigen presentation alone is not sufficient for T cell activation. A second immunoregulatory signal, a so-called checkpoint molecule, is necessary (41, 42). These molecules either have activating (i.e., co-stimulatory) or inhibiting (i.e., co-inhibitory) effects. Co-stimulatory markers promote T cell activation, proliferation, and differentiation; co-inhibitory markers inhibit T cell functioning and activation (43–45).

In tumors and various chronic infectious diseases, checkpoint molecules play important roles in regulating the immune response (10, 18). For example, in these diseases, PD-1 is upregulated. This results in the inhibition of immune cell activity so that pathological cells can hide from the immune system (46). This finding has already been applied in tumor therapy. For example, anti-PD-1 antibodies are used to treat melanomas and non-small cell lung cancer (11, 47).

Recently, soluble forms of checkpoint molecules were also found. In contrast to checkpoint molecules on the cell surface, they are less well studied and their role in immune regulation is not well understood. They can be generated *via* expression of the soluble form or by the cleavage of membrane-bound proteins by immune cells or tumor cells (21, 48, 49). There are no robust data for the correlation between the concentration of the soluble markers in synovial fluid or serum with their expression on the surface of immune cells. However, it is assumed that the stronger the surface expression of the markers, the greater the number of soluble markers found in synovial fluid.

In this study, we found that the mean concentrations of soluble immunoregulatory markers were generally lowest in control joints, TJA joints generally had lower concentrations than AIF joints, and PJI joints tended to have the highest concentrations, while sCD27, sBTLA, and sCD80 were the exceptions.

Our study revealed a significant difference for sCD80 comparing the CO and TJA groups, with higher mean concentrations in the TJA group. sCD80 is expressed by monocytes and B cells and is generated by alternative splicing (49). Whereas Kakoulidou et al. found an inhibitory effect on lymphocyte reactions and T cell proliferation, various other studies could describe an enhancement of T cell proliferation and IFN-γ production (21, 49–52). Furthermore, Haile et al. showed that a soluble form of CD80, CD80-Fc, was more effective in preventing the coinhibitory effect of the PD-1/PD-L1 pathway and in restoring T cell activation in comparison to blocking either PD-1 or PD-L1 with antibodies (52). Overlaying this finding with our data, the increased concentration of sCD80 in the TJA group compared to the CO group supports the idea that the prosthesis might activate the local immune system in the joint.

CD80 is expressed by macrophages, which are known to play an important role in aseptic loosening. Particles abraded from the prosthesis activate macrophages and promote osteoclast differentiation (53). This leads to local bone loss, which, in turn, can lead to aseptic loosening of the implant. In our data set, AIF patients showed a higher mean concentration of sCD80 than any other group and reached a statistical significance of < 0.001 when comparing AIF patients with the CO group. These findings suggest a role of macrophages and sCD80 in aseptic loosening. Furthermore, these data also suggest that blocking sCD80 to reduce an activation of the immune system activation could be a possible therapeutic option.

Another interesting soluble checkpoint molecule in our study is sCTLA-4. This marker presented the highest mean concentration in the PJI group and reached a significance < 0.001 when comparing the PJI and CO groups. sCTLA-4 is released by Treg cells, monocytes and immature DCs (48). Higher levels were found during immune activation and in different autoimmune and inflammatory diseases such as lupus, autoimmune thyroiditis, myasthenia gravis, and celiac disease (54–57). Furthermore, in different tumors a high level of sCTLA-4 was associated with a poor prognosis (58, 59). It could be shown that sCTLA-4, similarly to CTLA-4, inhibits T cells. Blocking sCTLA-4 led to elevated levels of cytokines, especially IFN-γ (21, 60). In contrast, other studies found that sCTLA-4 inhibits the inhibitory effect of CTLA-4 on T cells (61, 62). One possible explanation for these contradictory findings might be that the effects of sCTLA-4 depend on the activation status of the involved cells. Whereas sCTLA-4 might inhibit the CD80-CD28 interaction on resting cells, it may inhibit the CD80-CTLA-4 interaction on activated T cells, thereby preventing the inhibition of T cells. In PJI, inhibitory effects on T cells might promote the persistence of the infection. Thus, blocking sCTLA-4 may possibly lead to T cell activation and proliferation and represent a possible therapy.

Based on our data, sCD28 could also be another therapeutic avenue. However, not much is known about this molecule in the current literature. sCD28 is expressed by T cells and increased in autoimmune diseases such as lupus, Sjögren’s syndrome, allergic asthma and SLE (63–65). There are, however, as yet no data regarding its mechanism of action.

Our analysis of sBTLA found a significant difference between the AIF with TJA group (p = 0.036). Gorgulho et al. could show a positive correlation of sBTLA and BTLA expression on the cell surface (66). Soluble BTLA is increased in sepsis (67, 68). Dong et al. and Bian et al. showed that sBTLA correlated with a poor prognosis in HCC and pancreatic adenocarcinoma (69, 70). Furthermore, it correlated with the risk of death in clear cell renal cell carcinoma patients (22). There are different hypotheses how sBTLA could regulate the immune system. One possibility is that sBTLA could competitively bind HVEM on antigen presenting cells. On the other hand, it’s plausible that sBTLA mimics the inhibitory effect of sCTLA-4 (66). Thinking along this line, the elevated sBTLA in our study could regulate the immune system activation in aseptic loosening. Therefore, targeting this molecule might be useful in the therapy of AIF.

Differentiation between aseptic and septic loosening (which are related to AIF and PJI, respectively) is a big problem in everyday clinical practice; currently, neither clinical nor laboratory parameters can be used to clearly differentiate them (26). Various diagnostic tools, such as the MSIS criteria, are currently being used to diagnose PJI (2, 71). However, there is still no proper gold standard for diagnosis. The distinction between aseptic and septic joint inflammation is vital for subsequent treatment. In cases of AIF, the aseptically loosened prostheses can generally be immediately exchanged. PJI must typically be addressed several times (31). For the patient, PJI also means taking antibiotics and experiencing chronic health complaints (31). Comparing the mean concentration of the PJI and AIF group in this study, the PJI group showed higher levels. However, using the Kruskal-Wallis test, no significant difference between the two groups could be shown. Therefore, it could not be found a useful biomarker to differ a PJI from an AIF.

As mentioned earlier, PJI is usually a chronic infection. The role of immunoregulatory markers has already been investigated multiple times in other chronic diseases (9, 10, 72). In chronic infectious diseases such as HIV or HCV infections, co-inhibitory molecules, e.g., PD-1, show increased concentration (73, 74). Based on previous findings and the current findings, the assumption arises that these co-inhibitory markers play an important role in the immune response during PJI. By inhibiting the immune system, they pave the way for chronic disease. The infection persists within the joint and is not effectively resolved. Targeted blocking of these co-inhibitory markers using antibodies might neutralize the inhibition of the immune system. The infection may then be better addressed by the subsequent increased immune response and thus, satisfactorily treated.

However, one important next step for better diagnostic and therapeutic approaches utilizing the biology of soluble checkpoint inhibitors is not only a better understanding of their expression and mechanism of action but also how reproducible they are across different patient cohorts with differences in age, medication, site of surgery or comorbidities.

To address a potential influence of the site of surgery, we compared the level of checkpoint molecules of hip aspirates in comparison to knee aspirates. No significant trends could be found, except for sLAG-3 in the PJI group. If such an effect can be confirmed in a larger cohort, this would open up a very interesting discussion about differences of the local immunological microenvironment and how this could be used for targeted therapy.

As mentioned before, other comorbidities also need to be investigated in more detail. In this study, 10 patients out of 99 patients presented a RA. Previous publications could show that the levels of soluble immune checkpoints, such as sCD28, sCTLA-4, sCD80, and sPD-1 were higher in RA patients compared to patients without RA (75, 76). In our study, there was no significant difference between patients with and without RA. It is possible that the effect of an implanted protheses, aseptic loosening or PJI on the immune system might influence the expression profile of checkpoint molecules and disguise the influence of the RA. However, the value of this subgroup analysis is very limited due to the few numbers of RA patients. A significantly larger cohort is needed to allow for sufficient statistical power to perform meaningful statistics on the effect of RA. We also investigated whether DM type 2 had an influence on the investigated markers but could not find significant differences. It is noteworthy that most patients with DM type 2 were in the PJI group. This could be explained by the fact that patients with DM type 2 are prone to infections, especially periprosthetic joint infections (77, 78). In addition, DM type 2 is associated with obesity in the context of the metabolic syndrome and patients with a higher BMI have a higher risk for infections (77–79). These subgroup analyses imply that checkpoint molecules are not or only slightly influenced by the site of surgery, RA or DM type 2; however, such analyses have to be repeated in larger (multi-center) studies to clearly verify the comparability of patient groups.

Future studies should continue to investigate the roles of soluble immunoregulatory markers in aseptic and septic joint inflammation. There is hope that this could result in new diagnostic and therapeutic approaches for aseptic and septic joint inflammation. Targeted inhibition of particular markers with antibodies may also influence the chronic course of PJI and result in successful eradication of the infection.

In summary, this study demonstrates that the concentrations of the analyzed immunoregulatory molecules varied between control, TJA, AIF, and PJI joints. Ultimately, this study suggests that immunoregulatory markers, such as sBTLA (AIF vs. TJA) or sCD28 and sCTLA-4 (PJI vs. TJA), could be useful for diagnostic purposes as well as to develop new therapeutic approaches for AIF and PJI.

## Data Availability Statement

The original contributions presented in the study are included in the article/**Supplementary Material**. Further inquiries can be directed to the corresponding author.

## Ethics Statement

The studies involving human participants were reviewed and approved by the ethics committee of the University of Bonn, Germany. The patients/participants provided their written informed consent to participate in this study.

## Author Contributions

All authors listed have made a substantial, direct, and intellectual contribution to the work, and approved it for publication.

## Funding

This work was supported by a grant from the National Multiple Sclerosis Society (RG 1809-32591 to FS).

## Conflict of Interest

The authors declare that the research was conducted in the absence of any commercial or financial relationships that could be construed as a potential conflict of interest.

## Publisher’s Note

All claims expressed in this article are solely those of the authors and do not necessarily represent those of their affiliated organizations, or those of the publisher, the editors and the reviewers. Any product that may be evaluated in this article, or claim that may be made by its manufacturer, is not guaranteed or endorsed by the publisher.
