# Supplementary material for: sCD28, sCD80, sCTLA-4, and sBTLA Are Promising Markers in Diagnostic and Therapeutic Approaches for Aseptic Loosening and Periprosthetic Joint Infection
Source: Front Immunol. 2021 Aug 6;12:687065. doi: 10.3389/fimmu.2021.687065 (PMC8377391; doi:10.3389/fimmu.2021.687065)
Supplement: Supplementary file 1 [file DataSheet_1.docx]

**Supplementary tables**

**Supplementary table 1. Comparison of hip and knee aspirates**

The Mann-Whitney-U-test was used to evaluate the statistical significance of the differences between hip and knee aspirates. The level of significance was set at p < 0.05. There was a significant difference for sLAG-3 in the PJI group. **CO** = control group; **TJA** = total joint arthroplasty; **AIF** = aseptic implant failure; **PJI** = periprosthetic joint infection; **CTLA-4** = cytotoxic T-lymphocyte-associated protein 4; **PD-1** = programmed cell death protein 1; **PD-L1** = programmed cell death 1 ligand 1; **PD-L2** = programmed cell death 1 ligand 2; **TIM-3** = T-cell immunoglobulin and mucin-domain containing-3; **LAG-3** = lymphocyte-activation gene 3; **BTLA** = B- and T-lymphocyte attenuator; **HVEM** = herpesvirus entry mediator; **IDO** = indolamin-2,3-dioxygenase; **GITR** = glucocorticoid-induced TNFR-related protein; **CD** = cluster of differentiation.

| **marker** | **group** | **hip vs. knee** | **marker** | **group** | **hip vs. knee** |
| --- | --- | --- | --- | --- | --- |
| **sCTLA-4** | CO | p = 0.727 | **sCD27** | CO | p = 0.811 |
|  | TJA | p = 0.865 |  | TJA | p = 0.609 |
|  | AIF | p = 0.531 |  | AIF | p = 0.955 |
|  | PJI | p = 0.746 |  | PJI | p = 0.765 |
| **sPD-1** | CO | p = 0.161 | **sGITR** | CO | p = 0.937 |
|  | TJA | p = 0.286 |  | TJA | p = 0.201 |
|  | AIF | p = 0.569 |  | AIF | p = 0.955 |
|  | PJI | p = 0.091 |  | PJI | p = 0.466 |
| **sPD-L1** | CO | p = 0.217 | **sCD137** | CO | p = 1.000 |
|  | TJA | p = 0.919 |  | TJA | p = 0.541 |
|  | AIF | p = 0.392 |  | AIF | p = 0.691 |
|  | PJI | p = 0.558 |  | PJI | p = 0.860 |
| **sPD-L2** | CO | p = 0.692 | **sCD80** | CO | p = 0.217 |
|  | TJA | p = 0.759 |  | TJA | p = 1.000 |
|  | AIF | p = 0.649 |  | AIF | p = 0.459 |
|  | PJI | p = 0.399 |  | PJI | p = 0.062 |
| **sTIM-3** | CO | p = 0.469 | **sCD28** | CO | p = 0.209 |
|  | TJA | p = 1.000 |  | TJA | p = 0.865 |
|  | AIF | p = 0.733 |  | AIF | p = 0.865 |
|  | PJI | p = 0.110 |  | PJI | p = 1.000 |
| **sLAG-3** | CO | p = 0.217 | **IDO** | CO | p = 0.371 |
|  | TJA | p = 0.812 |  | TJA | p = 0.609 |
|  | AIF | p = 0.691 |  | AIF | p = 0.733 |
|  | PJI | **p = 0.028*** |  | PJI | p = 0.191 |
| **sBTLA** | CO | p = 0.937 | **sHVEM** | CO | p = 0.469 |
|  | TJA | p = 0.708 |  | TJA | p = 0.319 |
|  | AIF | p = 0.459 |  | AIF | p = 0.733 |
|  | PJI | p = 0.638 |  | PJI | p = 0.432 |

**Supplementary table 2. Comparison of patients with and without diabetes mellitus type II**

The Mann-Whitney-U-test was used to evaluate the statistical significance of the differences between patients with and patients without diabetes mellitus (DM) type II. The level of significance was set at p < 0.05. There was no significant difference for any soluble immunoregulatory marker. **CO** = control group; **TJA** = total joint arthroplasty; **AIF** = aseptic implant failure; **PJI** = periprosthetic joint infection; **CTLA-4** = cytotoxic T-lymphocyte-associated protein 4; **PD-1** = programmed cell death protein 1; **PD-L1** = programmed cell death 1 ligand 1; **PD-L2** = programmed cell death 1 ligand 2; **TIM-3** = T-cell immunoglobulin and mucin-domain containing-3; **LAG-3** = lymphocyte-activation gene 3; **BTLA** = B- and T-lymphocyte attenuator; **HVEM** = herpesvirus entry mediator; **IDO** = indolamin-2,3-dioxygenase; **GITR** = glucocorticoid-induced TNFR-related protein; **CD** = cluster of differentiation.

| **marker** | **group** | **DM vs. non-DM** | **marker** | **group** | **DM vs. non-DM** |
| --- | --- | --- | --- | --- | --- |
| **sCTLA-4** | CO | p = 0.667 | **sCD27** | CO | p = 0.308 |
|  | TJA | p = 0.457 |  | TJA | p = 0.898 |
|  | AIF | p = 0.797 |  | AIF | p = 0.587 |
|  | PJI | p = 0.715 |  | PJI | p = 0.440 |
| **sPD-1** | CO | p = 0.308 | **sGITR** | CO | p = 0.462 |
|  | TJA | p = 0.196 |  | TJA | p = 0.355 |
|  | AIF | p = 0.957 |  | AIF | p = 0.587 |
|  | PJI | p = 0.190 |  | PJI | p = 0.834 |
| **sPD-L1** | CO | p = 0.462 | **sCD137** | CO | p = 1.000 |
|  | TJA | p = 0.966 |  | TJA | p = 0.787 |
|  | AIF | p = 0.652 |  | AIF | p = 0.587 |
|  | PJI | p = 0.092 |  | PJI | p = 0.715 |
| **sPD-L2** | CO | p = 0.769 | **sCD80** | CO | p = 1.000 |
|  | TJA | p = 0.689 |  | TJA | p = 0.514 |
|  | AIF | p = 0.797 |  | AIF | p = 0.406 |
|  | PJI | p = 0.063 |  | PJI | p = 0.072 |
| **sTIM-3** | CO | p = 0.769 | **sCD28** | CO | p = 0.833 |
|  | TJA | p = 1.000 |  | TJA | p = 0.457 |
|  | AIF | p = 0.797 |  | AIF | p = 0.652 |
|  | PJI | p = 0.528 |  | PJI | p = 0.944 |
| **sLAG-3** | CO | p = 0.769 | **IDO** | CO | p = 0.154 |
|  | TJA | p = 0.230 |  | TJA | p = 0.698 |
|  | AIF | p = 1.000 |  | AIF | p = 0.797 |
|  | PJI | p = 0.333 |  | PJI | p = 0.081 |
| **sBTLA** | CO | p = 0.308 | **sHVEM** | CO | p = 0.923 |
|  | TJA | p = 0.698 |  | TJA | p = 0.514 |
|  | AIF | p = 0.797 |  | AIF | p = 0.464 |
|  | PJI | p = 0.200 |  | PJI | p = 0.255 |

**Supplementary table 3. Comparison of rheumatoid patients with non-rheumatoid patients**

The Mann-Whitney-U-test was used to evaluate the statistical significance of the differences between rheumatoid arthritis (RA) patients and non-rheumatoid patients. The level of significance was set at p < 0.05. There was no significant difference for any soluble immunoregulatory marker. **CO** = control group; **TJA** = total joint arthroplasty; **AIF** = aseptic implant failure; **PJI** = periprosthetic joint infection; **CTLA-4** = cytotoxic T-lymphocyte-associated protein 4; **PD-1** = programmed cell death protein 1; **PD-L1** = programmed cell death 1 ligand 1; **PD-L2** = programmed cell death 1 ligand 2; **TIM-3** = T-cell immunoglobulin and mucin-domain containing-3; **LAG-3** = lymphocyte-activation gene 3; **BTLA** = B- and T-lymphocyte attenuator; **HVEM** = herpesvirus entry mediator; **IDO** = indolamin-2,3-dioxygenase; **GITR** = glucocorticoid-induced TNFR-related protein; **CD** = cluster of differentiation.

| **marker** | **group** | **RA vs. non-RA** | **marker** | **group** | **RA vs. non-RA** |
| --- | --- | --- | --- | --- | --- |
| **sCTLA-4** | CO | p = 0.885 | **sCD27** | CO | p = 0.789 |
|  | TJA | p = 0.553 |  | TJA | p = 0.315 |
|  | AIF | p = 0.531 |  | AIF | p = 0.465 |
|  | PJI | p = 0.560 |  | PJI | p = 0.450 |
| **sPD-1** | CO | p = 0.561 | **sGITR** | CO | p = 0.588 |
|  | TJA | p = 0.301 |  | TJA | p = 0.545 |
|  | AIF | p = 0.875 |  | AIF | p = 0.564 |
|  | PJI | p = 0.274 |  | PJI | p = 0.833 |
| **sPD-L1** | CO | p = 0.361 | **sCD137** | CO | p = 0.468 |
|  | TJA | p = 0.163 |  | TJA | p = 0.534 |
|  | AIF | p = 0.551 |  | AIF | p = 0.347 |
|  | PJI | p = 0.599 |  | PJI | p = 0.962 |
| **sPD-L2** | CO | p = 0.423 | **sCD80** | CO | p = 0.271 |
|  | TJA | p = 0.201 |  | TJA | p = 0.235 |
|  | AIF | p = 0.917 |  | AIF | p = 0.835 |
|  | PJI | p = 0.867 |  | PJI | p = 0.529 |
| **sTIM-3** | CO | p = 0.423 | **sCD28** | CO | p = 0.568 |
|  | TJA | p = 0.584 |  | TJA | p = 0.475 |
|  | AIF | p = 0.175 |  | AIF | p = 0.754 |
|  | PJI | p = 0.450 |  | PJI | p = 0.450 |
| **sLAG-3** | CO | p = 0.410 | **IDO** | CO | p = 0.574 |
|  | TJA | p = 0.438 |  | TJA | p = 0.259 |
|  | AIF | p = 0.754 |  | AIF | p = 0.143 |
|  | PJI | p = 0.401 |  | PJI | p = 0.644 |
| **sBTLA** | CO | p = 0.779 | **sHVEM** | CO | p = 0.773 |
|  | TJA | p = 0.643 |  | TJA | p = 0.343 |
|  | AIF | p = 1.000 |  | AIF | p = 0.462 |
|  | PJI | p = 0.966 |  | PJI | p = 0.423 |
